# Stakeholders' Perceptions of the Roles and Regulations of Advanced Practice Nursing and Advanced Practice Midwifery

**DOI:** 10.1155/jonm/7475260

**Published:** 2025-10-24

**Authors:** Constance S. Shumba, Benard Daniel Mutwiri, Denis Munene, Rachel W. Kimani, Beatrice May, Isabel Kambo, Colette Henderson, Irene Mageto, Daniela Lehwaldt, Eunice Ndirangu-Mugo

**Affiliations:** ^1^The Aga Khan University School of Nursing and Midwifery East Africa, Aga Khan University-Kenya, Nairobi, Kenya; ^2^Institute for Health & Equity, Medical College of Wisconsin, Milwaukee, Wisconsin, USA; ^3^Neurosciences and Behavior, The Rockefeller University, New York, New York, USA; ^4^School of Health Sciences Dundee, University of Dundee, Dundee, UK; ^5^School of Nursing Psychotherapy and Community Health Dublin, Dublin City University, Leinster, Ireland

**Keywords:** advanced nursing and midwifery practice, qualitative research, roles and regulations, stakeholder perceptions

## Abstract

**Background:**

Advanced practice nursing (APN) and advanced practice midwifery (APM) in Kenya are critical in healthcare system strengthening by expanding access to quality care, particularly in underserved regions. Globally, the International Council of Nurses (ICN) defines APN as a level of nursing practice that requires a minimum of a master's degree, along with advanced clinical training and competency. Similarly, APM involves midwives with advanced training to provide specialized maternal and neonatal care. In Kenya, the APN and APM concepts were launched in 2020 and are still nascent, and their practice is determined by the context as provided by the country credentialing the practice. Despite the strides made in developing the advanced roles, there is an eminent gap in understanding stakeholders' perceptions of the roles, scope of practice, and regulation.

**Aim:**

To explore stakeholders' perceptions of the roles and regulations of APN and APM in Kenya.

**Design:**

An exploratory qualitative study was undertaken as part of a larger formative research conducted between January 2022 and November 2023, utilizing a multimethod design that included a gap analysis of the scopes of practice for APN and APM in Kenya. Data collection was conducted among purposively sampled participants using a total of 7 focus group discussions (FGDs), with three of them conducted among 14 APN and APM students and 4 FGDs among 16 members of the Kenyan-based nursing and midwifery associations. Seven key informant interviews (KIIs) were also held with 2 medical doctors (OBGYN), two faculty, and 3 students (practicing APN). A thematic analysis approach was employed, and reporting was done according to the Standards for Reporting Qualitative Research.

**Findings:**

Participant's perspectives were reflected in 5 themes: definition of APN/APM and scope; entry qualifications into APN/APM practice; roles of APN/APM; barriers to enacting APN/APM roles; the national policy and regulatory landscape for advanced practice roles.

**Conclusion:**

There were varied viewpoints on how APNs/APMs are defined in the Kenyan context. Similarly, views on the entry level qualifications into APN/APM were diverse. Many participants emphasized the importance of licensure, autonomy of practice, ability to manage patients independently, training, and competency. Well-defined policies and regulations can create an enabling environment for practice.

## 1. Background

The world continues to experience changes in the burden of disease with a growing prevalence of chronic diseases [1]. To achieve WHO's sustainable development goal 3 of attaining health for all at all ages, the health workforce must be aligned to promote health equity across the health continuum for a range of conditions [2, 3]. Advanced practice nurses (APNs) and advanced practice midwives (APMs) are an integral part of the health workforce that is delivering high-quality health services to populations across several countries, positively impacting health outcomes [4–6]. In settings across sub-Saharan Africa, such as Kenya, the health contexts are also evolving in line with the other global regions. The role of APNs and APMs in Kenya in driving progress towards universal healthcare coverage and cost-effective healthcare has been highlighted [7, 8]. Within the Kenyan context, universities have commenced educating APN and APM, and consequently, the issues of developing the scopes of practice and regulation of APN and APM have garnered interest among stakeholders [8].

To shape conversations, it is imperative to understand the definitions of APN and APM. There have been conflicting definitions of APNs and NPs across different contexts, yet it is important that stakeholders have clarity on the definition of APNs [9, 10]. APNs are registered nurses who have acquired advanced training and certification through further studies such as a master's degree or a doctorate in nursing and possess an advanced knowledge base, intricate decision-making abilities, and clinical competencies suitable for expanded practice [11]. APNs are particularly referred to as advanced practice registered nurses in the United States and form part of advanced practice providers (APPs) in healthcare that also includes physician assistants [12].

Notably, the critical point of definition is on skills, knowledge, and expertise, all of which are founded on advanced minimum training of a master's degree in nursing. ICN, however, notes that the practice of APNs is determined by the context as provided by the country credentialing the practice [11]. In other words, as much as ICN provides the basic definition of APNs, contextual differences exist from one country to another, and thus, the need to assess stakeholders' knowledge on the matter of scope, roles, responsibilities, and regulation of APN [13]. Note that different countries operationalize advanced practice roles very differently, and thus, two or more roles may exist in one country but with differing varying requirements and regulation compared to another country. Other countries have the same roles but use titles to refer to the roles, for example, nurse practitioner (NP), clinical nurse specialist (CNS), nurse specialist (NS), and APN to imply similar roles. There has never been an international consensus on definition of APM, but as with the APN, an APM is a midwife who has acquired further specialization training through additional education such as master's and doctorate degrees or their equivalents with emphasis on advanced knowledge and critical thinking skills to provide optimum care [12]. The International Confederation of Midwives [14] defines a midwife as a person who has completed a midwifery education program and is licensed to provide preventive, promotive, and detection of complications during pregnancy, labor, and the postpartum period.

There are also global variations concerning the scope of practice of AMP and the regulations guiding the same. A global SWOT analysis adduced that while APN is expanding globally, inadequate regulation undermines its potential impact [15]. In Kenya, the absence of formal APN policies limits role recognition and deployment [15]. In the UK, the Royal College of Midwives and the Nursing and Midwifery Council (NMC) formerly UKCC Statutory Midwives Committee maintained that APM or “higher level practice” was not justifiable as regular practicing midwives (PMs) practice at a specialist or advanced level the moment they register into the profession [16]. Currently, in 2024, the NMC has approved the regulation of ANP and AMP [17]. In Canada, there are well-developed competencies for entry-level midwives and “advanced level” midwives, but there is no consensus about what constitute advanced midwifery practice and as such it is regulated at the provincial territories not nationally [16]. In contrast, Ireland has a well-defined framework that guides the accreditation of advanced midwifery practitioners with formal recognition of APMs by the Nursing and Midwifery Board of Ireland [18]. Applicants for APM must be registered midwives with a minimum of a master's degree in a midwifery-related and relevant field. They must also have at least 7 years of postregistration experience, including 5 years in their chosen area of specialization in practice.

In sub-Saharan Africa, South Africa serves as a reference, having introduced the concept of APM in the late 1990s to enhance the quality of midwifery services through increased education of the attendants [19]. However, APM noted that their practice was not commensurate to with their advanced midwifery skills as they were not optimally utilized, resulting in expressed loss of self-confidence, and overall lack of recognition, support, and space to demonstrate their expertise [19]. In Kenya, the concept of APN/APM was launched in 2020 and is emerging. The Aga Khan University, School of Nursing & Midwifery (AKU-SONAM) and Masinde Muliro University of Science and Technology (MMUST) are the institutions providing APN and APM Programmes in Kenya. Conventionally, it is expected that all the stakeholders should be adequately knowledgeable about the scope and regulation of advanced nursing and midwifery. When advanced midwifery practice is developed, launched, and commenced, a clear conception among the stakeholders is vital to advance the discipline, its practice and regulation for the optimum benefit of the society, avoid discordance within the profession, facilitate seamless implementation, and support the realization of legitimacy internally and externally [20, 21].

There are deficiencies in the understanding of APNs and APMs because of questions of clarity of role definitions, scope of practice, role boundaries, nomenclatures, and proficiency, thereby creating barriers to the realization of these roles [9, 12, 22–26]. Yet, role clarity is the foundation on which professional identity can be anchored and thus form a framework on which consistent and desired outcomes of ANP can be defined, monitored, evaluated, and measured [9]. It is evident that even in countries where APN and APM were launched decades ago, there is still an eminent gap in the understanding of the roles, scope of practice, and therefore regulation of APN and APM, providing a strong rationale for building evidence to effectively ensure that APN and APM roles are effectively implemented and optimized thereby contributing to supporting universal health coverage in Kenya.

## 2. Methods

### 2.1. Study Aim

The main objective of this study was to explore the stakeholders' perceptions of the APN/APM practice areas, roles, responsibilities, and regulations in Kenya.

### 2.2. Study Design

This was a qualitative study designed based on the findings of a gap analysis of the scopes of practice for APN and APM in Kenya [8]. This study was part of a larger formative research, which employed a multimethod design to inform the development of the APN and APM scopes of practice in Kenya between January 2022 and November 2023.

### 2.3. Study Population and Sampling

The target population for this study was the APN and APM students, nursing and midwifery associations, and educators. Purposive sampling was used to recruit 37 study participants with diverse perspectives (see Table 1). A total of 7 focus group discussions (FGDs) and 7 key informant interviews (KIIs) were held. Three of the FGDs were conducted among 14 APN and APM students at the AKU-SONAM and MMUST, both of which are the only institutions that have implemented the APN and APM master's program in Kenya. Four FGDs were conducted among 16 members of the Kenyan-based nursing and midwifery associations, namely, the Kenya Progressive Nurses Association and Midwives Association of Kenya. The seven KIIs were also held with 2 doctors (OBGYN), 2 faculty, and 3 students (practicing APN)

### 2.4. Data Collection and Management

Structured FGD guide and KII guides were developed based on the previous gap analysis covering existing opportunities, challenges, and gaps relative to the APN and APM roles and responsibilities in the Kenyan context. The FGDs took one hour, while the KIIs lasted about 45 minutes. All the qualitative interviews were done virtually via a Zoom link that was shared by the research assistant to the study participants. The interviews were also recorded via an audio recorder, and later, the audios were transferred to a laptop. The audios were transcribed verbatim in word documents to form the 14 transcripts

### 2.5. Ethical Considerations

Ethical approval for this study was sought from the Aga Khan University Institutional Review Board before initiating the research (ISERC#-2021/IERC-146(v2) and NACOSTI/P/22/15755 to authorize execution of this research. Study details were fully explained, and informed consent was obtained from all participants prior to the FGDs and interviews. All data and transcripts were deidentified to uphold confidentiality, and study materials were stored in line with the AKU research data protection policy.

### 2.6. Data Analysis

NVivo software was used to analyze the qualitative data collected from the study participants. The seven FGD transcripts and seven KII transcripts were imported into NVivo software and analyzed using thematic content analysis [27]. A thematic approach was utilized to categorize the findings into items relative to the specific APN and APM roles and responsibilities, informing the scope of practice. The transcripts were then categorized into themes and subthemes, and reporting was done according to Standards for Reporting Qualitative Research (SRQR) [28]. The analysis findings from the qualitative phase were anticipated to provide a comprehensive framework of understanding the perceived roles, responsibilities, entry qualifications, and regulations of the APN and APM from the stakeholders' standpoint

## 3. Results

### 3.1. Demographics

The key themes and subthemes are shown in Table 2 with illustrative quotes.

A shown in Table 2, participants' perspectives were reflected in 5 themes: definition of APN/APM and scope; entry qualifications into APN/APM practice; roles of APN/APM; barriers to enacting APN/APM roles; the national policy and regulatory landscape for advanced practice roles.

### 3.2. Theme 1: APN/APM Definition and Scope

The study findings reveal that there are varied viewpoints on how APNs/APMs are defined in the Kenyan context. Many participants emphasized the importance of licensure, autonomy of practice, ability to manage patients independently, training, and competency.

“…But for the practice, if you are really practicing as a nurse, you're looking forward to have that independent practicing license that I'm accountable to what I'm doing. I'm accountable to my practice. That's what we're looking forward for.” PRACTICING KII 1

Autonomy of practice was highlighted as a key part of the APN/APM role characterized by being able to manage patients and then refer those they cannot manage.

“I feel like the ideal scope of practice for APN in Kenya should be the one where we as advanced practitioners are allowed to practice on our own. That is, we need to be given a list for us to be enabled to work a role, to have autonomy in our professional work. So, we need to have a scope of practice where we have the autonomy of practice, and we are also able to channel patients with those conditions which are unmanageable to by us to another levels.” STUDENT FGD 2

Relatedly, it was noted that the APN/APM has the training and competency attained at the master's level to enable them to practice independently.

“Advanced practice nurse is a nurse who has already attained a graduate degree that is master's degree in an area of specialization where they can be able to practice, I would say, independently.” FACULTY KII 1

Participants see APNs/APMs as having specialized knowledge and skills at a “mastery level,” enabling them to be consultants and experts in their fields of practice. Clinical practice and research are commonly identified roles, as are teaching, mentoring, and educating other nurses.

“This one is an education aspect of it, this is someone who has specialized and actually gone to that level of mastery of a certain area. It could be medical, surgical, it could be oncology, it could be midwifery. So, this is a nurse who has actually gone in terms of education level to a mastery level and even higher to a doctorate or a PhD level, but on one level of practice and this makes her to have an extra skill or an upper advantage.” ASSOCIATION FGD 2

### 3.3. Theme 2: Entry Requirements for APN/APM

The findings reveal diversity in perspectives on the educational prerequisites for entry into APN/APM practice. Many assert that a bachelor's degree should be the minimum, while others emphasized needing at least a master's degree.

“So, I would say that a nurse who wants to do the APN should have a degree that is the BSN, BSCN.” STUDENT FGD 1

“I believe in Kenya we're saying for one to practice as an advanced practice nurse one has to be at the master's at postgraduate level.” ASSOCIATION FGD 4

“…for them to do advanced nurse practitioner, they need to have done the bachelor's degree.” PRACTICING KII 1

“Also doctorate in nurse practice.” STUDENT FGD 1

There were a few who still see diploma-level education as a satisfactory foundation.

“I would want to look at the diploma level to be the basic. So, there are those ones who are going for higher diploma for specific areas, let's say critical care, nursing, oncology, whatsoever, they have advanced from the basic diploma.” ASSOCIATION FGD 1

### 3.4. Theme 3: Perceived APN/APM Roles

Most of the participants had a good understanding of the expected roles of APNs/APMs. The most identified roles are diagnosis, treatment, prescribing medications, and referring patients. APNs/APMs are seen as having an expanded scope that enables them to comprehensively manage patients. Clinical practice and research are commonly identified roles, as are teaching, mentoring, policy-making, educating nurses, and taking on leadership/management positions.

“…coming up with the right diagnosis and also prescribing medication…” PRACTICING KII 1

“Their scope of practice is quite broader than the other nurses. So, they can be able to treat.” STUDENT FGD 3

The area of teaching and mentoring was also cited as one where APN/APM could make significant contribution.

“Another competency as far as nursing education is concerned is I should be able to exhibit very good teaching and learning methodologies so that we use the various teaching and learning methods to ensure that I take care of all the students or all the learners. Bearing in mind that we have different types of learners, I should be able to also know the different teaching approaches to be able to give the best to the students so that we bring up the, we bring up quality students who are able to take care of the community.” STUDENT FGD 2

“Even a nurse who is able now to teach other nurses. They can also teach.” ASSOCIATION FGD 4

“And most of the other time they are mentors, they mentor students in the institution or in the department, like in maternity, they are the mentors, and they are mostly the planners of the patient care. They are able to detect what the patients are doing, and they are able to give the best care to the patient.” ASSOCIATION FGD 3

Additionally, it was noted that they have an important role in research and knowledge management:

“They should have some skill in research and what we call knowledge management. Research is part of those courses that they go through and in terms of knowledge management there is a lot of information that they need to have on evidence-based practice, evaluation of programs, interventions, critical thinking all those.” FACULTY KII 1

Participants also highlighted APN/APMs' roles in policy development, leadership, and management:

“I will emphasize on the policy skills, whereby for the advanced practice to improve its outcome, we should have that skill of able to contribute and develop in development of the healthy policy. Also, with the current trends in terms of nursing and health.” STUDENT FGD 2

“Leadership in health care was not in the hands of nurses. But now we've seen this evolvement that nurses are able to hold these key positions in leadership and management. I would say to begin with midwifery summation where I say a midwife I mean, advanced midwifery practice, I think to me, I feel it is that level of midwifery practice at which a midwife is able to use their expertise in management and care of patients that is clinical care. Anyone who does maybe an advanced level, I would say maybe those ones who do their master's in nursing or whatever specialty. They still have to leave the bedside and go to the management. This one for the managerial positions for the advanced nurse practitioners like they can manage institutions, they can be the CEOs they can be monitored even to be the CS of health and various similar positions.” STUDENT FGD 3

“The heights are rising right now towards practice and education in nursing and midwifery, and we can see a lot of involvement of our nurses and midwives in leadership and key positions, which is something that never used to exist. I remember in the early 90s to 2000s, Leadership in health care was not in the hands of nurses. But now we've seen this evolvement that nurses are able to hold these key positions in leadership. Her as a leader should be able to see and to this that both supplies and equipment and everything that is put in use. The nurses are well-acquainted to all the machinery and equipment, and they are able to execute their mandate as nurses.” ASSOCIATION FGD 1

### 3.5. Theme 4: Barriers to Enacting APN/APM Roles

Participants identified several barriers that hinder the enactment of APN/APM roles in Kenya. Among them, devolution has created challenges, as county governments under the devolved system prefer hiring diploma nurses, leading APNs/APMs to conceal their credentials due to fear of unemployment.

“…the other one is that the devolution was a blessing but also a threat to the nursing profession and especially the masters level why because most of the governors they want the nurses who are hands on, certificate level, diploma level so even those ones who have done the master's program they are hiding their master's certificates, sometimes even the undergraduate certificates, because if you have it, you will not be employed. So, you'd rather use the diploma one in order to sustain yourself or be able to get your salary than to expose yourself that you've done the APN or APM. So, devolution which has been useful in those other areas is also a threat to this particular program because the nurses who come out may not be able to practice independently the way that we thought or be able to freely express, share their skills at that higher level in the areas of work.” FACUTY KII 1

Additionally, role confusion between different nursing cadres also exists.

“In Kenya, the way we have started, I think there's a lot of confusion as yet, because you find like what you're saying about a nurse practitioner at a very rudimentary and primary level, it's done by the nurses, general nurses who exist.” ASSOCIATION FGD 4

There is also resistance from doctors who perceive APNs/APMs as competitors.

“…they feel like those advanced practitioners, if they are allowed to study, they keep their own clinics, they could be a threat because they can do the deliveries more than them so of course and the senior cadre, we are really, really be against. In terms of management, of course, they appear in administration and management circles will not be very, very happy.” OBS/GYN KII 3

Limited mentors to supervise APNs/APMs and few universities offering the needed training programs also impede advancements in this field.

“Then the other challenge that is there is about mentorship. When you look into this program, we lack mentors who should help us, rather who should help the nurse practitioners to learn the ropes and maybe provide some practical and emotional support? If you look into professions like the medicine, you find that the senior most, the consultant doctors, they are always there with the juniors to walk the road, to walk the journey with them. Now these nurse practitioners lack mentorship.” STUDENT FGD 3

There are also a limited number of universities offering APN/APM, so the courses are not widely accessible:

“…those outside there who will be interested to be APNs but when it comes to learning institutions, you find that they are limited. Like now we can say there are only two learning institutions. That's Masinde Muliro and Aga Khan.” STUDENT FGD 2

### 3.6. Theme 5: Policies and Regulations

Most participants emphasized the importance of well-defined policies and regulations to enable APN/APM practice. Revising the Public Health Act to formally acknowledge APN/APM qualifications is identified as a crucial step.

“Career progression, that one should also be changed, and the Public Health Act should also be changed to incorporate nurse as an entity into that. Because when we read that one, we see only public health and the nursing bit is missing. And yet we are also the key players in promotive and preventive health services.” ASSOCIATION FGD 4

Recommendations include developing scopes of practice, and schemes of service aligned with qualifications.

“I would strongly recommend that the nursing council recognizes the APM and provides license for them. Also, they should develop a scope of practice and a scheme of service for the same and of course also include them in the nursing and we do free policy.” ASSOCIATION FGD 2

Further, participants noted the importance of developing policies to formally recognize APN/APMs and to enhance training and practice:

“I'm saying the policies that can be put in place to ensure the APN, APN is allowed to practice in our country, is the one, the policies that comes or addresses the issues on the qualifications and the entry.” PRACTICING KII 2

“Policies and regulation necessary to enhance adoption of APN. I think nurses or nurses who are practicing clinics nurse practice or qualified nurses should be included or should be, the voices should be in health or MOH policy makers so that they will be able to sensitize the policy makers about the roles of APNs and the resourcefulness of APNs in the country and health sector. So, I think nurses or APNs prepared nurses should be able to forge head to be an advocate of the profession in the MOH wherever we are especially when the national government, MOH, already recognized the role, it will be able to include the roles in their healthcare management system so that the public and the structures of healthcare will be able to be recognized. So, I think it's a human resource policy, one, leadership, health care leadership, management policies. APN's voices should be heard or seen because they will be the one to advocate for the APN roles in the country.” STUDENT FGD 1

Involving different stakeholders like the Nursing Council of Kenya as well as national/county governments is deemed important.

“… like the regulatory bodies who are supposed to help this nurse who has gained her master's or her PhD to be able to qualify and become an advanced nurse practitioner or an advanced midwife practitioner in that field and give them all the rights and mandates to work under that jurisdiction. So, I believe also the government has a role to play to make sure through the government policies. I believe also changing the policies and regulations of our country can be able to help and maybe enable and be able to revert the challenges that are already there or have already been because we are trying to evolve.” ASSOCIATION FGD 2

“It is the nursing council that needs to hold the hands of this profession and take it to the highest level where this program can be recognized and be put as an Act of Parliament that should be tabled that would allow these nurses to practice independently.” FACULTY KII 1

Additionally, standardizing training sponsorship policies was also suggested:

“The other thing we need to maybe encourage the policy of sponsorship. When maybe sometimes we find that when the county allocates some funds for those who will be given funding to advance. Most of the time it may go to one cadre. So, it should be a policy that it should be standardized. They should standardize and say, this number should go. So, nurses, this number, clinical officer, we say this number, doctors, this number. So, the sponsorship or the donation or the funding to be a crosscutting.” ASSOCIATION FGD 3

## 4. Discussion

### 4.1. Contextual Definition of the APN and APM Scope

The findings of this study revealed that there are varied viewpoints on how APNs/APMs are defined in the Kenyan context, with the contextual APN/APM definition of scope explained in terms of expectations of licensure, autonomy of practice, training and competency, and specialization at the master's level. The findings are consistent with the ideal scope of practice, which emphasizes factors such as expectations of licensure, autonomy, training, competency, and specialization at the master's level [25, 26, 29]. The APN is expected to maintain a high degree of professional autonomy and competence to engage in clinical decision-making, perform evaluations, provide diagnoses and prescriptions, assume responsibility for case management, and contribute to the planning and implementation of programs and care plans [30]. Similarly, Poghosyan et al. [31] emphasized that nurses aspire to an ideal scope that involves the diagnosis and management of acute and chronic illnesses, indicating a desire for increased autonomy. Autonomous practice is considered as independent and self-directed which therefore means that an individual APM is responsible and accountable to their own practice [32]. This perspective begs the question of standardization because if an individual APM is self-directed in the provision of health service, there is no guarantee that all the service will comply or align with any existing standards of operation. Elliot et al. [32] noted that independence and self-direction should be understood in terms of ability based on skills, experience, and knowledge.

### 4.2. Entry Qualifications for APN/APM

This study noted varying perceptions on the entry requirements into advanced practitioner roles with a combination of undergraduate, postgraduate, or diploma education within nursing. Other authors have asserted that the entry requirements for APN/APM vary [20, 33], with some advocating for a common identity and uniform entry requirements while others argue for multiple routes of education [34, 35]. When considering the required educational prerequisites for enrolling in the APN/APM course, most participants agreed that having a bachelor's degree was crucial, which is consistent with findings by [25]. Specific requirements for APN and APM include a Bachelor of Science in nursing or equivalent qualifications for APN and midwifery training at the advanced diploma level for APM, as is the case in Botswana [36]. The 2004 American Association of Colleges of Nursing's position statement on doctoral preparation for advanced practice is widely regarded as a seminal directive that continues to shape debates on APN education in the United States. While it signified a classical benchmark in envisioning doctoral entry, master's-level preparation remains the pragmatic norm. In contrast, researchers argue that globally, educational standards for APN are heterogeneous, with considerable variation in entry requirements and regulatory frameworks, highlighting the persistent challenge of achieving international harmonization [25]. Some participants emphasized the significance of a Diploma course as a foundational step for growth in nursing and midwifery, in agreement with Worthington [37], who highlighted the importance of clinical experience and basics of nursing in APN training and harmonization of entry grades for a nursing diploma in Kenya, allowing students to grow through the ranks.

### 4.3. APN/APM Roles

Participants in the study demonstrated familiarity with the roles of APNs and APMs. Their responses highlighted tasks related to prescribing, diagnosing, treating, referring patients, and managing complications. This corresponds to findings on patient management and prescribing respectively [25, 38]. Further the results reinforce those of a previous study highlighting that APN and APM practitioners are perceived as expert clinicians, educators, researchers, and leaders [39–42]. They can diagnose and treat patients, prescribe medications, provide counseling, and coordinate care. Moreover, they play a crucial role in health promotion, disease prevention, and patient advocacy. Roles such as teaching and mentoring were perceived aspects of APN and APM, aligning with other findings emphasizing nurses' involvement in education and research [43]. Study participants stressed the fundamental role of research in clinical practice for nurses and midwives, reinforcing findings pointed out by [44] who highlighted the importance of research among APNs. The APN role encompasses the integration of research, education, care practice, and management. According to [32], the research skills of APMs should imply that they are knowledgeable in research, can do their independent research, and collaborate with others in conducting research, being involved in research projects, and making use of research in advancing practice of midwifery to provide holistic and evidence-based care. On this basis, the APMs can serve as educators, consultants, change agents, and auditors although this should not be confused that APMs' roles focus shifts to academics than midwifery.

There was agreement from our study participants that APN and APM play a crucial role in shaping health policy development. This view is supported by [45] who found that APN leaders were actively involved in shaping healthcare policies through their participation in committees, task forces, and consultations. Further, participants acknowledged that advanced practice nurses and midwives should take leadership and management roles which is consistent with other studies [46, 47]. The leadership domain of APM's scope of practice provides that action of APMs should be fine-tuned to also focus on providing both clinical and professional leadership [48]. Expertise, on the other hand, is a function tied to the expectation that an AMP is competent and highly experienced in advanced midwifery which encompasses an extended knowledge base and possess large-scale knowledge of midwifery, higher level thinking, and critical evaluation extending beyond labor ward [49].

### 4.4. Barriers to Enacting APN/APM Roles

Several barriers to the enactment of APN and APM in Kenya were considered by the participants including minimal recognition by County governments which prefer to employ nurses with lower levels of qualifications. The situation is such dire that some of the APN/APM practitioners hide their real qualifications to secure job placements with government bodies. This observation reverberates with the findings of other studies; for example, in the United States, fewer APRNs are adequately recognized, and as such, they are denied certain privileges [50]. Although the trend has been changing in the United States, the most recent data still put the statistics of APRNs with admissions privileges at 43% although it is not clear why majority of the APRNs are denied such privileges [51].

Our study participants commented on the lack of understanding and recognition of their scope of practice. According to a study by Banaser et al. [52], misconceptions about APN and APM roles among healthcare providers and the general public hinder their full utilization which has a significant impact on healthcare delivery.

The confusion of roles between APN/APMs and physicians is equally a significant concern as noted by other studies with roles such as diagnosis and prescription having been traditionally domiciled in the hand of physicians [53]. There is thus confusion on whether APN/APMs can fully replace physicians or complement them or not all. This is coupled with the controversy in the title of APN/APMs based on the specialty roles they play following their advancements [53]. The confusion is particularly pronounced in settings where the APN/APMs are privileged to admit, diagnose, and make prescriptions because in some sense, those are roles conventionally preserved for physicians [54]. Holanda and her colleagues in their study of “practice profile and competencies” of APNs and APMs in Belgium further adds that the confusion of role is the main reason behind wrong deployment of APN or APM in roles that they might not be adequately competent to execute and perform [55].

Finally, the shortage of APN/APM mentors coupled with the limited number of higher education institution offering APN/APM courses poses a barrier to the advancement of the programs [17]. For instance, in sub-Saharan Africa, the evidence from the data shows that unlike other programs in most health professions education, APN/APM is gradually gaining popularity and as such has minimal visibility among the public and potential students or health professionals who might want to advance their studies and practice. An assessment of oncology practices in academic institutions and their affiliated hospitals in the United States noted that there existed a gap due to limited access to research, training, and support for APN/APMs [58]. Tailored mentorship programs for APN/APM graduates are supported by a range of studies, highlighting the need for intense involvement of a protégé with an expert researcher for optimal scholarly productivity and demonstrating the positive impact of mentorship on nursing competency and the successful implementation of formalized mentorship programs [59, 60]. Our study findings indicate that there are a limited number of universities in Kenya offering APN and APM which is consistent with previous findings [61].

### 4.5. Policies and Regulations

The successful development and implementation of APN and APM require a policy to guide its development, flexible, and visionary educational curricula and appropriate regulatory standards [62]. The space in which APN/APMs learn, qualify for practice, and enter practice is generally characterized with a lot of regulatory and policy vacuums. The result is a lack of direction, roadmap, and provisions on which the programs can be promoted in higher education institutions. This situation creates a dilemma in defining the scope and roles of APNs/APMs within public healthcare systems [63], particularly as there are no clear-cut regulation frameworks and policies which recognize and define the scope of practice of APN/APMs, nor define how they should be absorbed in the healthcare, leadership, research, and education. The global regulation of APNs by national governments, professional bodies, or local employers is highlighted, with the Netherlands exemplifying a validated, protected title accredited by the Accreditation Organization of Netherlands and Flanders (NVAO) [26].

In fact, the lack of the policies and regulations is one of the major barriers to the progressions of APN/APMs in some parts of the world [64]. In countries where there are clear regulations, for example, USA and Ireland, the APN/APM has registered significant progress [30]. Notably, the regulatory framework is ambiguous from one country to the other. The author of [65] noted that in countries like Sweden, Australia, and United Kingdom (UK currently but NMC Council approved additional regulation for advanced practice on 27th March 2024), the regulations of nurses and regulations of APNs are assumed to be the same. In other words, same legislation is used for nurses and APNs and prompting the handling of APN just like other nurses without the same qualification, implying that the scope, practice, and accountability of APNs is regulated by the same legislation regulating the scope, as registered nurses. Although the International Council of Nurses (ICNs) have made their declaration on the position and possible guidelines and regulation particularly on APNs, the same creates provisions to accommodate variations in the healthcare systems from country to country [11]. Consequently, standardization remains a challenge. The regulation of APN/APM is a complex and varied issue, with significant differences in scope, advanced practice training, and regulation across different countries [22]. The implementation of the Advanced Practice Framework in Wales has been highlighted as a potential solution, providing a clear career development structure and ensuring consistency in clinical practice skills [66]. These findings underscore the need for a more standardized and consistent approach to the regulation of APN/APM.

### 4.6. Strengths and Limitations

The qualitative research on stakeholders' perceptions of APN and APM in Kenya offers valuable insights into the roles and regulatory environment of these professions. The strength of this study lies in its comprehensive approach, utilizing a multimethod design, which informed the qualitative phase that incorporates FGDs and KIIs with a diverse range of participants, including students, medical doctors, faculty, and members of nursing and midwifery associations. This diversity enhances the depth and breadth of the findings, reflecting a wide array of perspectives. The identification of five major themes provides a structured understanding of the key issues surrounding APN and APM in Kenya, from definitions and entry qualifications to the roles, barriers, and regulatory landscape. However, the study has limitations. The purposive sampling technique, while beneficial for targeting specific groups, may introduce bias and limit the generalizability of the findings. Additionally, the relatively small sample size and the focus on Kenyan stakeholders might not capture the full range of experiences and opinions that could be present in different regions or contexts. Despite these limitations, the study contributes significantly to the discourse on advanced practice roles in nursing and midwifery, highlighting the need for clear policies and regulatory frameworks to support these roles in Kenya.

## 5. Conclusions

Our study established significant variations on the contextual definition of the scope, but the key components in terms of a distinctive scope for APN and APM were found to be a master's specialization, training and competency, expectation of licensure, and autonomy of practice. In the context on the definition of scope, the study thus concludes that APN/APM are identifiable with roles including diagnosis, prescription, treating, referring patients, managing complications as well as leadership in research and education. Additionally, the study found that APN/APMs are expected to take leading roles in teaching and mentorship particular to nurses and/or midwives in training or internship and policy-making in healthcare systems and capacity building. While exploring the basic entry qualification into APN/APM, the study established mixed responses. That notwithstanding, bachelor's degree in nursing or midwifery and master's degree in the same are considerably the basic qualification that a regular nurse or midwives should have to advance to APN/APM. The study further concludes that although significant progress in being made, barriers including role confusion, fear of competition from physicians, limited advancement opportunities in local universities, limited mentorship, minimal or nonrecognition by potential employers, and limited privileges given to APNs/APMs in the healthcare system pose the greatest encounters to the progressions of APN/APM. Finally, the study concludes that there exists a vacuum in the legislative and regulatory landscape in terms of laws, policies, and guidelines is a matter which complicates the launch, uptake, and progression of APN/APM in many sub-Saharan Countries and particularly in Kenya.

### 5.1. Implications for Nursing and Policy

Based on the conclusions of the study, it is imperative to establish initiatives that would see the development, launch, uptake, and absorption of APN/APM rise in Kenya and similar settings. Among the initiatives which should be considered as a matter of urgency include a comprehensive definition of the scope of APN/APM are as provided for by the ICN and ICM. Since the provisions of ICN/ICM are widely researched and consulted from across the globe, aligning local definitions of scope to theirs would ease possible tensions and contentions as to what should or should not be accommodated in the scope of practice of APN/APM. Besides a clear and comprehensive definition of the scope of APN/APM, it is imperative to equally define the role of APN/APMs as provided for by ICN/ICM with professionally agreeable variations as would be acceptable in the global context. Clearly detailed roles would help address the fears among some physicians regarding perceived competition from APN/APM. It is crucial to identify the roles which qualified APN/APM should undertake in delivering healthcare whether in the public or private settings or social care. Such roles should be captured in regulations and policies [25]. Nevertheless, the quality of training should be certified and accredited by both local and internationally recognized health organizations including ICN and should be monitored to ensure that the actual training of APN/APM graduates qualifies and adequately prepares them to take up the roles defined.Furthermore, the study recommends that the minimum entry qualification into APN/APM should be consultatively developed and standardized so that all universities offering the programs publish the same qualification and admit students based on the qualification criteria. The minimum entry qualification should therefore induce the need to establish policies and legislative framework for the implementation of APN/APM in Kenya. Since APN/APM is a new phenomenon in Kenya, there is urgent need to develop relevant policies and regulatory frameworks including passing an Act of Parliament providing for recognition of the two disciplines (APN and APM), policies and regulation on the scope of practice, professional roles of the same, and policies and regulation of the two courses in higher health educations institutions. This should be coupled with efforts to popularize the provision of APN/APMs in higher education institutions in Kenya and establish mentorship programs to inspire the concept.

## Figures and Tables

**Table 1:** Distribution of the study participants.

Study participant	Organization	Data collection	1st round	2nd round	Total
1. Associations	KPNA	FGD	3	4	7
	MAK	FGD	4	5	9

2. Students still in university	AKU	FGD	4		4
	MMUST	FGD	6	4	10

3. Practicing APN	AKU	KII	3		3

4. Obs/gynae	KNH	KII	2		2

5. Faculty	MMUST	KII	2		2

Subtotal					37

**Table 2:** Themes, subthemes, and sample quotations identified in interviews with participants.

Theme	Subtheme	Illustrative quotes
APN/APM definition and scope	Licensure; autonomy of practice; training and competency	“I feel like the ideal scope of practice for APN in Kenya should be the one where we as advanced practitioners are allowed to practice on our own. That is, we need to be given a list for us to be enabled to work a role, to have autonomy in our professional work…” STUDENT FGD 2
Entry qualifications	Doctorate; masters; bachelor's; diploma	“I believe in Kenya we're saying for one to practice as an advanced practice nurse one has to be at the master's at postgraduate level.” ASSOCIATION FGD 4
APN/APM roles	Diagnosis; treatment; prescribing medication; referring patients; research; teaching and mentoring; policymaking; leadership and management	“Coming up with the right diagnosis and also prescribing medication.” PRACTICING KII 1
Barriers to enacting APN/APM roles	Preferential hiring; role confusion; resistance from other cadres; limited mentors; limited universities	“…they feel like those advanced practitioners, if they are allowed to study, they keep their own clinics, they could be a threat because they can do the deliveries more than them so of course and the senior cadre, we are really, really be against. In terms of management, of course, they appear in administration and management circles will not be very, very happy.” OBS/GYN KII 3
Policies and regulations	Policy; scheme of service; policies for enhanced training and practice; involvement of government and other stakeholders; standardized sponsorship	“… the regulatory bodies who are supposed to help this nurse who has gained her master's or her PhD to be able to qualify and become an advanced nurse practitioner or an advanced midwife practitioner in that field and give them all the rights and mandates to work under that jurisdiction… I believe also changing the policies and regulations of our country can be able to help…” ASSOCIATION FGD 2

## Data Availability

The data that support the findings of this study are available from the corresponding author upon reasonable request.
